# Virginia Society of Dental Surgeons

**Published:** 1846-12

**Authors:** S. M. Shepherd

**Affiliations:** Secretary pro tem.


*Virginia Society of Dental Surgeons.*—We take the following synopsis of the doings of the Virginia Society of Dental Surgeons from the Petersburg Republican.—*Eds.*

This Society, according to appointment, held its fourth annual meeting in the town of Petersburg, on the 15th of October, 1846. As soon as a quorum had convened, the vice-president, John G. Wayt, called the meeting to order, and S. M. Shepherd was appointed secretary. After receiving reports, &c., a letter was presented from Dr. A. D. Lewis, containing his resignation as a member of the Society, on the ground that he had removed from Virginia.

Ordered that the resolution of the last meeting, respecting members who have not paid their dues, shall not be in force until after the next annual meeting; and that such members be furnished with a copy of said resolution in the interim.

The executive committee recommended Dr. R. H. Sedwick as a suitable candidate for membership, and he was unanimously elected, and received the Society's diploma. The election of officers for one year then took place, and the following was the result:

S. Lethbridge, *President,* Richmond,
John G. Wayt, *Vice-president,* do.
James D. McCabe, *Cor. and Rec. Secretary,* Abingdon.
S. M. Shepherd, *Treasurer,* Petersburg.

*Executive, Examining and Publishing Committee.*

W. W. H. Thackston, Farmville, T. B. Hamlin, Wytheville,
John McConnell, Richmond, J. W. Shepherd, Petersburg,
W. C. Crump, Richmond.

The following resolutions were presented and passed:

By S. M. Shepherd.—In view of the present state of the public mind on the subject of dental surgery—the great want of dental literature of that kind which would be most likely to show the utility of dentistry, *Resolved,* That it is advisable for this society to publish a periodical which shall be devoted to that object.

By John McConnell.—*Resolved,* That each and every member of this society furnish an essay, calculated to enlighten the public mind on the subject of dentistry, and submit it to the next meeting for adoption and publication.

*Resolved,* That the town papers be requested to publish a synopsis of the proceedings of this meeting.

Dr. Thackston then read the annual address, which was ordered to be published in pamphlet form, for distribution among the members of the society.

The next annual meeting of the society will be held on the first Wednesday in October, 1847, in the city of Richmond.

S. M. SHEPHERD, *Secretary pro tem.*

